# Echocardiographic Evaluations in Sick Neonates in Neonatal Intensive Care Unit: A Clinical Study

**DOI:** 10.7759/cureus.53054

**Published:** 2024-01-27

**Authors:** Kushal Desai, Amar Taksande

**Affiliations:** 1 Pediatrics, Jawaharlal Nehru Medical College, Wardha, IND

**Keywords:** neonates, respiratory distress, neonatal intensive care unit, echocardiography, sick newborn

## Abstract

Introduction

Echocardiography is pivotal in neonatal care by providing valuable insights into cardiac function, anatomy, and hemodynamics. The ability of echocardiography to guide clinical decision-making is evident in its capacity to influence and change management strategies. Therefore, the goal of the current study was to know the prevalence of heart disease and the association between echocardiographic indications and neonatal outcomes and interventions.

Materials and method

This prospective cross-sectional study was conducted in the Neonatology Department, Datta Meghe Institute of Higher Education and Research, Sawangi, Wardha. Ill neonates admitted to the neonatal intensive care unit (NICU) were selected in a randomized manner, and echocardiography was performed. The decision was made on the indications for echocardiography, the echocardiographic findings, and any modifications to the clinical care. Mean differences were compared using an unpaired Student's t-test. A significant level is defined as a p-value of less than 0.05.

Results

Of all the ill neonates, heart disease was present in 60 (52.6%) neonates. The most frequent indication for echocardiography was respiratory distress in 39 (34.21%). In neonates with heart disease, 27(45%) had acyanotic heart disease, nine (15%) had cyanotic heart disease, and 24(40%) had miscellaneous heart disease. The mean appearance, pulse, grimace, activity, and respiration (APGAR) score at five minutes in neonates without heart disease was 9.05, and for those with heart disease, it was 8.95, with no statistically significant difference. Out of 114 neonates, the mean NICU stay of neonates without heart disease was 6.59 days, and those with heart disease was 9.95 days with a p-value of 0.0001, hence showing statistically significant differences. Out of all the 114 neonates, 57.89% underwent no intervention, 36.85% underwent medical intervention, and 5.26% underwent surgical interventions. Out of 114 neonates, 101 neonates were discharged, and 13 neonates were dead. Out of the 54 neonates with no cardiac abnormality, 50 (92.59%) were discharged, and four (7.41%) neonates were dead. Out of the 60 neonates with cardiac abnormality, 51 (85%) were discharged, and nine (15%) were dead, with a p-value of 0.203, showing no statistical significance between cardiac abnormality and neonatal outcome.

Conclusion

Our study emphasizes the pivotal role of echocardiography in the NICU for evaluating ill neonates. The findings underline the significance of early detection and precise diagnostic insights provided by echocardiography, contributing to tailored management strategies. The study highlights the transformative impact of echocardiography on clinical decision-making, facilitating timely interventions and improving overall neonatal care. Echocardiography is essential to improving outcomes for these susceptible newborns in the NICU as we continue to expand our knowledge of neonatal heart health.

## Introduction

In the NICU, clinicians have been using echocardiography since the 1990s. Initially, pediatric cardiologists primarily performed these echocardiographic studies to diagnose congenital heart diseases (CHDs) and screen for patent ductus arteriosus (PDA). However, in recent years, neonatologists have grown interested in using echocardiography to assess infant hemodynamic instability [1]. The widespread availability and miniaturization of echocardiography technology have led to an increased use of this diagnostic tool in neonatal intensive care units (NICUs) worldwide. Its function is swiftly evolving, with neonatologists employing it to inform clinical decisions concerning unwell infants. Echocardiography allows for a direct assessment of hemodynamics at the bedside and serves as an extension of the clinical examination, aiding in evaluating cardiovascular well-being in critically ill infants. The physiological information obtained can guide interventions based on the underlying pathophysiology [2].

Echocardiography is employed for various purposes in the neonatal intensive care setting, including diagnosing pulmonary hypertension, assessing patent ductus arteriosus, evaluating hemodynamics, gauging cardiac function, and identifying conditions like pericardial effusion and cardiac tamponade. While its popularity is on the rise, there is a notable absence of well-organized training programs for neonatologists to attain expertise in echocardiography [3]. When dealing with a newborn exhibiting cyanosis, respiratory distress, or a shock-like appearance, clinicians must consider various potential causes, including congenital heart disease, myocarditis, and issues related to the pulmonary, central nervous system, hematologic, infectious, and metabolic systems. While traditional methods like physical examination, electrocardiography, and chest X-rays may not reliably identify congenital heart disease in the early neonatal period, echocardiography provides immediate and detailed information about cardiac anatomy, aiding in the prompt differentiation of patients with significant congenital heart disease from others. In some cases, echocardiographic findings alone are unique and sufficient for an accurate diagnosis [4].

## Materials and methods

Study design, setting, and participants

This descriptive, cross-sectional study study involved all of the ill newborns in the NICU. It was carried out over two years in the Department of Pediatrics at Datta Meghe Institute of Higher Education and Research, Sawangi, Wardha.

Definition of Ill Neonates

Neonatal convulsions, asphyxia at birth and hypoxic-ischemic encephalopathy (HIE), respiratory distress, neonatal sepsis, preterm neonates, newborns born of traumatic or instrumental labor, metabolic problems with seizures, congenital malformation of the central nervous system, and neural tube defects are some of the high-risk neonates taken into account in this study.

Inclusion and Exclusion Criteria

Inclusion criteria comprised all ill neonates admitted to the NICU. Exclusion criteria consisted of full-term healthy neonates maintaining O_2_ concentration and neonates in NICU with hyperbilirubinemia only for phototherapy.

Ethics consideration and sample size

The Institutional Review Board of Datta Meghe Institute of Higher Education And Research, Wardha, approved the study protocol with no. DMIMS(DU)/IEC/2022/1071.

The formula used for the calculation of the sample size is n=[DEFF*Np(1-p)]/ [(d^2^/Z^2^_1-α/2_*(N-1)+p*(1-p)], where N means population size (for finite population correction factor), p means hypothesized % frequency of outcome factor in the population, d refers to confidence limits as a percentage of 100 (absolute +/- %) and DEFF means design effect.

Data collection

The assessment of factors that designate a newborn as an ill neonate involved obtaining a comprehensive maternal history and reviewing prenatal and postnatal data. Every unwell newborn admitted to the NICU was chosen non-randomized by the inclusion criteria. Residents in the neonatology department perform the echocardiography initially, in case of any unusual finding, the pediatric cardiologist is consulted. The morphology of the results was examined and noted, and a clinical link was made with other echocardiography findings. Once the equipment was ready, the baby was positioned on the examination table, typically on their back, with additional support provided by blankets or towels to maintain the desired position. Ultrasound gel was applied to the transducer to ensure optimal acoustic contact with the baby's skin, and the transducer was gently placed on the baby's chest. Adjustments were made to obtain specific heart views, including standard views such as the parasternal long-axis and short-axis, apical four-chamber, and subcostal views. Color Doppler was used during the examination to assess blood flow and identify abnormalities. Imaging settings were adjusted to optimize image quality, including depth, gain, and focus. Attention was paid to cardiac cycle timing to capture specific phases of the cardiac cycle. Images and video clips of relevant cardiac structures and functions were recorded, and any abnormalities or significant findings were documented for later review. After obtaining the necessary images, the healthcare professional conducting the echocardiogram reviewed the results to assess the structure and function of the heart. Findings were communicated with the healthcare team and the baby's caregivers, and post-procedure care included the removal of electrodes and gentle cleaning of the baby's skin to ensure their comfort and well-being. The entire process adhered to institutional protocols and guidelines for neonatal echocardiography. The prospective collection of information encompassed all echocardiograms conducted in the neonatal unit. The study involved identifying indications for performing echocardiography, assessing echocardiographic findings, and documenting any resultant adjustments in clinical management.

Statistical analysis

Statistical analysis, both descriptive and inferential, utilized Stata software (Stata 10, StataCorp LLC, College Station, USA). Quantitative data was examined through mean, median, and standard deviation, while qualitative data was summarized using percentages and proportions. Differences in proportions were compared using the Chi-square test and Fischer's exact test. Unpaired Student's t-test was employed for comparing means, with a significance level set at a p-value less than 0.05.

## Results

Out of 114 sick neonates admitted to the NICU, 54 neonates (47.4%) had normal cardiac anatomy, and 60 neonates (52.6%) had some structural or functional abnormality. The ratio of neonates with normal cardiac anatomy to neonates with structural or functional cardiac abnormality is 0.9 (Table 1). A higher percentage of ill neonates had some structural or functional cardiac abnormality.

**Table 1:** Prevalence of cardiac structural or functional abnormality in ill neonates in NICU NICU - neonatal intensive care unit

Sample size (ill neonates in NICU)	With normal cardiac anatomy	With structural or functional cardiac abnormality
114 (100%)	54 (47.4%)	60 (52.6%)

Out of 114 neonates, respiratory distress was present in 39 neonates, out of which 17 (43.59%) had normal cardiac anatomy, and 22 (56.41%) had a structural or functional abnormality, the most common indication for echocardiography. Indications for echocardiography in descending order were respiratory distress, asymptomatic murmur, birth asphyxia, not maintaining oxygen saturation (SpO_2_), prematurity, abnormal antenatal scan, intrauterine growth retardation, trachea-esophageal fistula, cyanosis, bounding pulse with tachycardia and apnea of prematurity (Table 2). The most common indication for echocardiography was respiratory distress.

**Table 2:** Indications for echocardiography

Indication for echocardiography	Heart disease absent (n=54)	Heart disease present (n=60)	Total (n=114)
Respiratory distress	17 (43.59%)	22 (56.41%)	39 (100%)
Asymptomatic murmur	15 (75%)	5 (25%)	20 (100%)
Birth asphyxia	5 (38.46%)	8 (61.54%)	13 (100%)
Not maintaining SpO_2_	0	11 (100%)	11 (100%)
Prematurity	5 (45.45%)	6 (54.55%)	11 (100%)
Abnormal antenatal scan	2 (40%)	3 (60%)	5 (100%)
Intrauterine growth restriction	3 (75%)	1 (25%)	4 (100%)
Tracheoesophageal fistula	3 (100%)	0	3 (100%)
Cyanosis	0	2 (100%)	2 (100%)
Bounding pulse with tachycardia	1 (50%)	1 (50%)	2 (100%)
Apnea of prematurity	2 (100%)	0	2 (100%)
Arrhythmia	1 (50%)	1 (50%)	2 (100%)

Out of 114 neonates, 27 had acyanotic heart disease, nine had cyanotic heart disease, and 24 had miscellaneous heart disease (patent foramen ovale [PFO], persistent pulmonary hypertension of the newborn [PPHN]). Out of the 54 neonates with cardiac abnormality, 24 neonates were preterm, 12 neonates were late preterm, and 24 neonates were term. Out of 54 neonates with cardiac abnormality, most frequent cardiac abnormality was PPHN (n=15). PFO, patent ductus arteriosus (PDA) with PFO, PDA, and significant PDA were present more in preterm neonates than in term neonates. Ventricular septal defect (VSD), transposition of great arteries (TGA), and PPHN were present more in term neonates than in preterm neonates (Table 3). Acyanotic heart disease was more common than cyanotic and miscellaneous groups of heart disease. Preterm and term neonates had more cardiac abnormality than late preterm. PPHN was the most frequent cardiac abnormality.

**Table 3:** Distribution of ill neonates according to gestational age and echocardiographic findings PDA - patent ductus arteriosus; VSD - ventricular septal defect; TAPVC - total anomalous pulmonary venous connections; PPHN - persistent pulmonary hypertension of the newborn; TOF - tetralogy of fallout; TGA - transposition of great arteries; PFO - patent foramen ovale

Classification of heart disease	Echocardiographic findings (n=54)		Preterm (n=24)	Late preterm(n=12)	Term (n=24)
Acyanotic heart disease (n=27)	PDA	14	7 (50%)	3 (21.43%)	4 (28.57%)
	PDA with PFO	6	4 (66.67%)	1 (16.67%)	1 (16.67%)
	VSD	5	0	1 (20%)	4 (80.00%)
	Significant PDA	2	2 (100%)	0	0
Cyanotic heart disease (n=9)	TAPVC	4	1 (25%)	1 (25%)	2 (50%)
	TGA	2	0	0	2 (100%)
	TOF	3	1 (33.33%)	2 (66.67%)	0
Miscellaneous (n=24)	PPHN	15	2 (13.33%)	2 (13.33%)	11 (73.33%)
	PFO	9	7 (77.78%)	2 (22.22%)	0

Out of 114 neonates, 101 neonates were discharged, and 13 neonates were dead. Out of the 54 neonates with no cardiac abnormality, 50 (92.59%) were discharged, and four (7.41%) neonates were dead. Out of the 60 neonates with cardiac abnormality, 51 (85%) were discharged and nine (15%) were dead (Table 4). A greater percentage of neonates with cardiac abnormality died. There was no significant difference between cardiac abnormality and neonatal outcome (p=0.203).

**Table 4:** Correlation of cardiac abnormality with neonatal outcome p=0.203

Heart disease present	Discharged (n=101)	Dead (n=13)	Total (n=114)
No	50 (92.59%)	4 (7.41%)	54 (100%)
Yes	51 (85%)	9 (15%)	60 (100%)

Out of 114 neonates, 101 neonates were discharged, and 13 neonates died. In neonates with normal anatomy (n=54), 50 neonates (92.59%) were discharged, and four neonates (7.41%) died. In neonates with trivial cardiac abnormality (n=29), 28 neonates (96.55%) were discharged, and one neonate (3.45%) died. In neonates with structural abnormality (n=16), 11 neonates (68.75%) were discharged, and five neonates (31.25%) died. In neonates with functional abnormality (n=15), 12 neonates (80%) were discharged, and three neonates (20%) died (Table 5). A greater percentage of neonates died of structural abnormality with statistical significance (p=0.019).

**Table 5:** Classification of heart disease vs outcome p=0.019

Classification of heart disease	Discharged (n=101)	Dead (n=13)	Total (n=114)
Normal anatomy	50 (92.59%)	4 (7.41%)	54 (100%)
Trivial cardiac abnormality	28 (96.55%)	1 (3.45%)	29 (100%)
Structural abnormality	11 (68.75%)	5 (31.25%)	16 (100%)
Functional abnormality	12 (80%)	3 (20%)	15 (100%)

Out of 114 neonates, the mean NICU stay with neonates without heart disease was 6.59 days with a standard deviation of 4.45 days, and those with heart disease were 9.95 days with a standard deviation of 4.13 days with a p-value of 0.0001. There is statistical significance in mean NICU stay in days in neonates with and without heart disease.

## Discussion

Congenital heart disease is the most prevalent developmental abnormality, and it stands as a primary cause of both neonatal mortality and morbidity. Delayed identification of critical congenital heart disease (CCHD) can result in cardiac failure, cardiovascular collapse, and heightened mortality if not promptly diagnosed. Critical congenital heart disease in newborns might manifest with clinically unnoticed low blood oxygen saturation. In recent years, there has been a notable shift in the role of echocardiography within NICUs. Neonatologists are increasingly incorporating echocardiography into their diagnostic toolkit for evaluating unwell newborns, and its utilization in NICUs is rapidly expanding.

Our study endeavors to determine the prevalence of structural or functional cardiac abnormalities in unwell neonates through echocardiography. Specifically, we assessed the correlation between indications for echocardiography and subsequent medical or surgical interventions, as well as investigated the relationship between the indication for echocardiography and the overall outcome of the neonates.

Table 6 depicts various studies related to the distribution of neonates according to the prevalence of heart disease. A study done by Whitehall et al. [5] reported that the prevalence of heart disease was 56.6.%. Subhani et al. [6] also conducted a study in which the prevalence of heart disease was 49.5%, and Arshad et al. [7] conducted a study where the prevalence of heart disease was 44.1%. Studies done by Whitehall et al., Subhani et al., and Arshad et al. [5-7] had a comparable prevalence to our present study, indicating 52.6 %. Whereas studies conducted by Moss et al. [8] and Kadivar et al. [4] showed a prevalence of heart disease to be 90.2% and 746 %, respectively (Table 7).

**Table 6:** Distribution of neonates according to the prevalence of heart disease in various studies

Author	Year of publication	Population	Neonates with heart disease	Prevalence
Whitehall et al. [5]	1999	537	304	56.6%
Moss et al. [8]	2003	82	74	90.2%
Kadivar et al. [4]	2008	241	180	74.6%
Arshad et al. [7]	2021	2729	1206	44.1%
Subhani et al. [6]	2022	212	105	49.5%
Present study	2024	114	60	52.6%

**Table 7:** Studies regarding the most common indication for echocardiography PDA - patent ductus arteriosus

Study	Year of publication	The most common indication for echocardiography
Moss et al. [8]	2003	Asymptomatic murmur (29%)
Kadivar et al. [4]	2008	Asymptomatic murmur (45%)
EL-Khuffash et al. [9]	2013	Assessment of PDA (51%)
Harabor et al. [10]	2015	Assessment of PDA (52.1%)
Hernández-Benítez et al. [12]	2016	Hemodynamic stability and sepsis (53.3%)
Papadhima et al. [11]	2018	Assessment of PDA (61%)
Present study	2024	Respiratory distress (34.21%)

Table 7 demonstrates various studies showing the most common indication for echocardiography. Studies conducted by Moss et al. [8] and Kadivar et al. [4] showed that the most common indication for echocardiography was an asymptomatic murmur present in 29% and 45% of neonates, respectively. Studies by El-Khuffash et al. [9], Harabor et al. [10], and Papadhima et al. [11] showed that the most common indication for echocardiography was to assess PDA which was present in 51%, 52.1%, and 61%, respectively. The present study depicted the most common indication to be respiratory distress (34.21%) and the second most common indication to be asymptomatic murmur (17.54%).

Table 8 shows various studies on how echocardiographic findings changed management in neonates. Studies done by Kadivar et al. [4] and Hernández-Benítez et al. [12] showed that echocardiographic findings changed management in 159 (66%) and 11 (73%) neonates, respectively. In the present study, the change of therapeutic management was done in 48 (42.1%) neonates, which has a comparable result to the studies done by Corredera et al. [13] and El-Kuffash et al. [9], where a change in management was done in 18 (37%) and 80 (40%) neonates, respectively. Khamkar et al. [14] and Papadhima et al. [11] had, based on echocardiography findings, a change in management in 74 (39.6%) and 267 (48%) neonates, respectively, which is also comparable to our study.

**Table 8:** Studies where echocardiographic findings changed management in neonates

Author	Year of publication	Population	Echocardiographic finding that changes therapeutic management in n (%)
Kadivar et al. [4]	2008	241	159 (66%)
Corredera et al. [13]	2014	50	18 (37%)
El-Kuffash et al. [9]	2013	199	80 (40%)
Khamkar et al. [14]	2015	187	74 (39.6%)
Hernández-Benítez et al. [12]	2016	15	11 (73%)
Papadhima et al. [11]	2018	599	267 (48%)
Present study	2024	114	48 (42.1%)

In our study, out of 114 neonates, no Intervention was done in 66 (57.89%) neonates, medical intervention was done in 42 (36.85%) neonates, and surgical intervention was done in six (5.26%) neonates. The most frequent intervention done was paracetamol plus fluid restriction.

Limitations

The limitations of this study are that it is a single-center study, and different NICUs may have variations in patient demographics, protocols, and resources, influencing the external validity of our findings.

## Conclusions

In conclusion, the evaluation of echocardiography in ill neonates in the NICU is paramount for understanding the prevalence of heart disease in this vulnerable population. Echocardiography serves as a crucial diagnostic tool with diverse indications, ranging from routine screening to assessing specific clinical presentations. The prevalence of heart disease in neonates emphasizes the significance of early and accurate diagnosis, allowing for timely intervention and improved outcomes. Ultimately, the integration of echocardiography into the comprehensive care of ill neonates in the NICU not only aids in the early identification of congenital or acquired heart diseases but also significantly impacts clinical management. This emphasizes the importance of continued research and advancements in echocardiographic techniques to enhance diagnostic accuracy and refine therapeutic strategies, ultimately improving the overall care and outcomes for neonates with cardiac issues in the NICU.
